# Exploring Tafamidis Effects Through PBPK–QSP Modelling

**DOI:** 10.3390/pharmaceutics18030367

**Published:** 2026-03-15

**Authors:** Seweryn Ulaszek, Bartek Lisowski, Barbara Wiśniowska, Sebastian Polak

**Affiliations:** 1 Chair of Pharmaceutical Technology and Biopharmaceutics, Faculty of Pharmacy, Jagiellonian University Medical College, Medyczna 9, 30-688 Kraków, Poland; seweryn.ulaszek@uj.edu.pl (S.U.); bartek.lisowski@uj.edu.pl (B.L.); 2 Doctoral School of Medical and Health Sciences, Jagiellonian University Medical College, 30-688 Kraków, Poland; 3 Department of Social Pharmacy, Faculty of Pharmacy, Jagiellonian University Medical College, Medyczna 9 Street, 30-688 Kraków, Poland; b.wisniowska@uj.edu.pl; 4 Certara Predictive Technologies, Certara UK Limited, Level 2-Acero, 1 Concourse Way, Sheffield S1 2BJ, UK

**Keywords:** transthyretin, tafamidis, quantitative systems pharmacology, physiologically based pharmacokinetics

## Abstract

**Background/Objectives**: Tafamidis, a transthyretin kinetic stabilizer, increases circulating transthyretin levels in treated patients. While this effect is well documented, its underlying mechanism remains incompletely understood. This study aimed to evaluate the performance of physiologically based pharmacokinetic (PBPK) model performance and to calibrate a hypothesis-consistent quantitative systems pharmacology (QSP) model of tafamidis and transthyretin dynamics to explore mechanistic hypotheses underlying the clinically observed increase in circulating transthyretin and the associated dose–response relationship. The PBPK model constitutes the primary framework, while the coupled QSP component illustrates how tafamidis exposure predictions can be used to evaluate mechanistic hypotheses of TTR turnover. **Methods**: A PBPK–QSP model was constructed in Simcyp (V23) using LUA-based modules. The PBPK part was parameterized from the literature and validated against data from therapeutic single-dose, therapeutic multiple-dose, and supratherapeutic dose clinical studies. The QSP part of the model describes tafamidis–TTR binding kinetics, stabilization, and clearance of bound complexes. Simulations were performed in thirty virtual healthy male subjects aged 30–40 years, incorporating physiological variability in baseline TTR concentrations. **Results**: Mean predicted versus observed ratios of tafamidis AUC and $C_{max}$ values were within a 1.3-fold range across validation studies. The integrated model reproduced the clinically reported 33% increase in TTR concentration through a calibrated clearance-scaling factor. It supports the hypothesis that reduced clearance of tafamidis-bound TTR may explain the observed effect without modifying TTR synthesis. Dose-sensitivity simulations indicated that patients with low baseline TTR may achieve adequate stabilization at reduced doses, while those with higher baseline TTR concentration may require higher doses. **Conclusions**: The developed PBPK–QSP model not only reproduces tafamidis pharmacokinetics and TTR responses but also proposes a plausible mechanistic hypothesis consistent with clearance modulation of stabilized TTR contributing to the clinical effect.

## 1. Introduction

Transthyretin amyloidosis (ATTR) is a progressive, life-threatening disorder caused by destabilization of transthyretin (TTR), a homotetrameric protein primarily synthesized in the liver [1,2]. Tetramer dissociation into monomers is the rate-limiting step of amyloid formation. Liberated monomers can misfold, aggregate, and deposit in tissues such as the myocardium and peripheral nerves, giving rise to ATTR cardiomyopathy (ATTR-CM) or polyneuropathy. The disease occurs in hereditary (ATTRv) or wild-type (ATTRwt) forms and is associated with substantial mortality. The introduction of kinetic stabilizers that bind to the thyroxine-binding sites of TTR and inhibit tetramer dissociation, such as tafamidis, has significantly improved outcomes in ATTR cardiomyopathy [3,4,5].

A consistent pharmacodynamic (PD) signature of tafamidis therapy is an increase in circulating TTR concentrations, typically around 33% above baseline in both ATTRv and ATTRwt. Although this rise is clinically reproducible, its mechanistic basis remains unresolved [6,7]. Stabilization reduces tetramer dissociation, but it is unclear whether, under physiological, non-mass-conserved conditions, this effect can fully account for the magnitude of the observed increase [8,9,10]. Therefore, mechanistic understanding of the origin of the TTR concentration rise requires models that incorporate both drug–protein binding and TTR turnover physiology.

Several TTR-focused modeling approaches have been proposed over the past decade. Subunit-exchange kinetic models accurately quantify tetramer dissociation under physiological conditions using in vitro or ex vivo plasma experiments [8]. Minimal turnover models couple synthesis, dissociation, reassociation, and degradation of TTR and show that slowed dissociation alone cannot reproduce the >30% clinical rise in circulating TTR without invoking additional mechanisms, such as altered clearance or changes in monomer disposition [9,10]. Population PK–PD models, most notably Tess et al. (2023), integrate in vitro binding, TTR concentrations, and clinical endpoints from the Transthyretin Amyloidosis Cardiomyopathy Clinical Trial (ATTR-ACT) to link tafamidis exposure with TTR stabilization and disease progression [5,11]. These analyses confirm near-complete stabilization at the approved 80 mg tafamidis meglumine dose and provide insight into the relationship between binding-site occupancy and clinical biomarkers.

Physiologically based pharmacokinetic (PBPK) modeling provides a mechanistic representation of drug disposition across organs and tissues, accounting for absorption, distribution, metabolism, and excretion based on anatomical and physiological principles [12]. Quantitative systems pharmacology (QSP) extends this to molecular and cellular processes underlying drug action (here, ligand–protein binding, tetramer stabilization, monomer generation and loss, and TTR turnover) [13]. An integrated PBPK–QSP model is therefore uniquely suited to translate tafamidis pharmacokinetics into mechanistic predictions of TTR homeostasis, offering the ability to formulate and test biological hypotheses and identify parameters that drive uncertainty.

As mentioned above, the study by Tess et al. established an important link between tafamidis binding and disease progression by modeling TTR concentrations and clinical endpoints as a function of steady-state tafamidis exposure [11]. While valuable, this approach relies on population PK rather than multiorgan PBPK, and thus does not capture tissue-level distribution, dynamic binding competition, or physiological constraints on tafamidis disposition. Additionally, the model proposed by Tess et al. explicitly assumes that monomer degradation greatly exceeds reassociation and fits several kinetic parameters simultaneously to reproduce the ~30% TTR increase observed after treatment initiation.

In contrast, the PBPK–QSP framework proposed in this manuscript incorporates experimentally supported parameters wherever possible, including human in vivo TTR turnover data and in vitro kinetic constraints from subunit exchange assays [8,14,15]. This reflects a key conceptual stance: mechanistic models must respect the origin and quality of parameter estimates, rather than expanding the parameter set to force agreement with a single clinical time point. Any sufficiently flexible model can be tuned to match sparse observations; explanatory value arises predominantly when a model reproduces data based on a physiological description, and not because its degrees of freedom are abundant. Our integration therefore makes explicit which physiological measurements, such as organ-level clearance, monomer internalization, and elimination pathways, remain critical gaps for better mechanistic understanding of tafamidis-modified TTR dynamics.

The primary aim of the present study is the development of a physiologically structured PBPK model of tafamidis that integrates currently available knowledge on its physicochemical properties, plasma protein binding, and systemic disposition. This PBPK framework is then coupled to a QSP module to illustrate how exposure predictions can be translated into mechanistic hypotheses regarding TTR turnover. In this work, we examine one biologically plausible hypothesis—that tafamidis binding modifies effective tetramer elimination—motivated by experimental observations of ligand-dependent TTR internalization and cellular processing. Importantly, the QSP component is intended as a hypothesis-testing extension rather than a uniquely identifiable mechanistic model. Alternative mechanistic explanations and their limitations have been analyzed previously, and the present PBPK–QSP framework is designed to be extensible as new data on TTR biology and tafamidis pharmacology become available [10].

In summary, the objective of this study was to develop and validate an integrated PBPK–QSP model of tafamidis kinetics and TTR turnover. The developed model (1) captures tafamidis pharmacokinetics across organs and tissues; (2) quantitatively describes drug binding, stabilization, and TTR turnover under physiological, non-mass-conserved conditions; (3) enables mechanistic hypothesis testing to evaluate which biological processes can plausibly account for the clinically observed increase in circulating TTR; and (4) identifies physiological and kinetic parameters that constrain our mechanistic understanding of TTR homeostasis under stabilizer therapy, thereby mapping explicit blind spots, i.e., parameters and processes we know we do not yet know well enough, to guide future experimental and clinical data collection.

## 2. Materials and Methods

### 2.1. Clinical Trial Data Used for Model Development and Validation

Clinical pharmacokinetic data from selected tafamidis studies were used to develop and evaluate model performance against observed human exposure across a range of dosing conditions. Study characteristics and data sources are summarized in Table 1.

Model accuracy was assessed using fold error, defined as the ratio of the predicted to the observed pharmacokinetic summary statistic (Predicted/Observed), and reported for the corresponding mean, median, or geometric mean values, as applicable. To provide a global assessment of model bias and precision, Average Fold Error (AFE) and Absolute Average Fold Error (AAFE) were additionally calculated using fold errors derived from exposure metrics (AUC and $C_{max}$) only, consistent with standard PBPK model evaluation practice. AFE was defined as the geometric mean of the Predicted/Observed ratios, calculated as $10^{mean(log(Predicted/Observed))}$, while AAFE was defined as the geometric mean of the absolute fold deviations, calculated as $10^{mean(|log(Predicted/Observed)|)}$. Model performance was interpreted against commonly applied PBPK acceptance criteria, with fold error values within 0.5–2.0 considered acceptable and values within 0.8–1.25 considered indicative of strong predictive performance.

### 2.2. PBPK Model Development

The general structure of the PBPK model was previously proposed and implemented in Simcyp Simulator (version 23) [20]. The model follows a whole-body, perfusion-limited PBPK framework in which all anatomical compartments are represented as well-stirred, kinetically homogeneous units, assuming instantaneous distribution within each tissue relative to inter-compartmental blood flow. Drug transport between compartments is governed by organ-specific blood flows and tissue-to-plasma partition coefficients.

The PBPK model-simulated plasma concentration of tafamidis is then used as an external, dynamic effect driving variable for the downstream QSP model. The simulated plasma concentration–time profile of tafamidis serves as a dynamic forcing function for the downstream QSP model, with no feedback from the QSP system to the PBPK component.

Overall, the present framework represents a hybrid PBPK–QSP model combining physiologically structured distribution and binding with empirically parameterized elimination and calibrated turnover scaling.

#### 2.2.1. Drug Physicochemical Properties and Blood Protein Binding

Tafamidis is a monoprotic acid with a pKa of 3.73 and molecular weight of 308.12 g/mol, with the molecular weight of tafamidis meglumine salt being 503.33 g/mol [21]. A logP value (3.91), calculated using the ALOGPS model, was used to parametrize the PBPK model [22]. The plasma unbound fraction of the drug has a value of 0.008 (0.8%). Apart from its affinity for TTR, which is a key element of its mechanism of action, tafamidis also exhibits high affinity for plasma albumin, consistent with interactions between a weakly acidic small molecule and proteins with basic binding domains [23]. Binding to plasma proteins is part of the developed QSP model, as described further in the QSP model development and Section 3.

#### 2.2.2. Dissolution and Absorption

Tafamidis has been reported as having low solubility and variable permeability; however, in vitro–in vivo extrapolation of Caco-2 permeability results suggests that it is actually a highly permeable drug, since its apparent permeability ($P_{app}$) equals $30.90\pm 3.28\times 10^{-6}\;\frac{m}{s}$ from the apical to basolateral side and $31.90\pm 2.82\times 10^{-6}\;\frac{m}{s}\;$ from the basolateral to apical side [24]. The above-mentioned $P_{app}$ value was used to estimate effective permeability ($P_{eff}$) using linear scaling, as described by Sun et al. [25].

**Figure A1:**
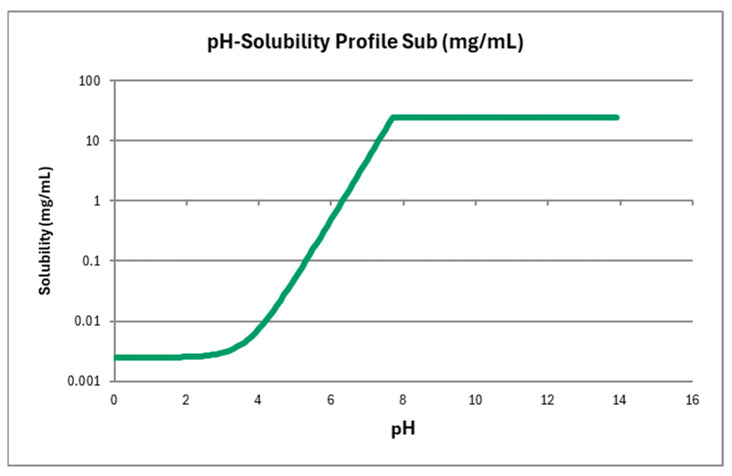
Predicted pH-dependent solubility profile.

According to regulatory data, tafamidis meglumine solubility at a pH 6.8 buffer is 3.121 $\frac{mg}{mL}$. It is also known that in an alkaline environment (0.1 M NaOH), solubility is higher than 4.19 $\frac{mg}{mL}$, while solubility in water is higher than 4.63 $\frac{mg}{mL}$ [26]. The presence of meglumine, an alkalizing agent, explains the higher solubility in pure water compared to the pH 6.8 buffer. In the developed PBPK model, tafamidis meglumine solubility was described as a pH-dependent parameter, incorporating the adjusted Henderson–Hasselbalch equation for prediction, based on the measured value at pH 6.8 [27]. Predicted intrinsic solubility, S_o_ = 0.0025 mg/mL, seems reasonable considering low solubility in low pH. The predicted pH-dependent solubility is presented in Figure A1. The commercially available tafamidis meglumine is manufactured as gelatine-encapsulated powder; thus, it is assumed that it is released rapidly once it enters the duodenum, allowing for the immediate start of the dissolution process and absorption. For description of the absorption process, the ADAM (Advanced Dissolution, Absorption, and Metabolism) model was used, with the stomach serving as the dose-accepting compartment and upstream driver of intestinal absorption. Fed and fasted conditions were handled implicitly within the Simcyp ADAM framework by applying condition-specific gastrointestinal physiology (including gastric emptying time, luminal fluid volumes, bile salt concentrations, and intestinal transit), as implemented in the Simcyp Healthy Volunteers population. The above-reported Caco-2 $P_{app}$ data were used to estimate $P_{eff}$ as $6.97\times 10^{-4}\frac{cm}{s}\;$ [28]. It was assumed that permeability is the same for each of the seven intestinal subcompartments, whereas local pH, fluid dynamics, and GIT transit time follow inter-individual variability as implemented in the Simcyp Healthy Volunteers population [20]. Additionally, the gastric emptying time varies between virtual individuals [29].

#### 2.2.3. Volume of Distribution and Tissue-to-Plasma Partitioning

The apparent volume of distribution after oral administration of tafamidis meglumine to healthy volunteers was reported as 16 L (0.2 L/kg for an 80 kg patient) [30]. For a full PBPK distribution model, the volume of distribution at steady state ($V_{ss}$), accounting for distribution in the following tissues: adipose, bone, brain, gut, pancreas, heart, kidney, liver, lung, muscle, skin, and spleen, was calculated following the Sawada equation [31].

Partition coefficients between each tissue and plasma ($K_{p}$)—except for bone, pancreas, muscle, and skin—were obtained from a rat PK study on tafamidis distribution, following the assumption that drugs are distributed in rats similarly to humans [24,32]. For prediction of $K_{p}$ for bone, pancreas, muscle, and skin, the Rodgers and Rowland model was incorporated as described previously [33]. The model estimated $V_{ss}\;$ as 13.53 L for an 80 kg patient (0.17 L/kg).

#### 2.2.4. Metabolism and Excretion

Tafamidis is metabolized primarily by UGT-mediated glucuronidation, predominantly by UGT1A1, UGT1A3, and UGT1A9 [34]. Unfortunately, no intrinsic clearance values or contributions of each of the enzymes can be found in the publicly available literature; thus, metabolism was described with a holistic parameter of oral clearance—0.263 L/h—as stated in drug-prescribing information [35]. Therefore, the model is not suitable for populations with altered UGT abundance (i.e., certain diseases, including liver cirrhosis) or for assessing drug–drug interactions, as it lacks the mechanistic scalability offered by intrinsic clearance data. Based on documentation submitted to the FDA, a large fraction of pharmacologically inactive tafamidis metabolite is expected to be excreted with bile; the proposed model does not account for this route of elimination, as there is not enough data to mechanistically describe such a process.

#### 2.2.5. Enzyme- and Transporter-Mediated Drug–Drug Interactions

In vitro data presented in the regulatory documents do not exclude inhibition of intestinal UGT1A1 and induction of CYP2B6 and CYP3A4. It has been suggested that tafamidis might also inhibit OAT1, OAT3, and BCRP transporters [34]. Nevertheless, interaction with enzymes and transporters is not relevant for this model, as drug–drug interactions (DDI) are not in the scope of this study.

### 2.3. QSP Model Development

Tafamidis drug action was described using a basic ligand–protein binding model assuming two binding sites on the TTR molecule. The model assumes that after tafamidis binding, TTR undergoes conformational changes as previously observed [36,37]. Since structural modifications might alter molecular properties, the bound protein is hypothesized to exhibit distinct attributes compared to its unbound form, making it less susceptible to clearance or more susceptible to undergoing tissue internalization [6,37]. This hypothesis is supported by experimental studies demonstrating ligand-dependent differences in transthyretin internalization and cellular handling following binding of distinct stabilizers [38]. In the model, kinetically stabilized TTR is assumed to have a negligibly small dissociation rate on the timescale of plasma turnover. This reflects near-complete stabilization observed at therapeutic tafamidis exposure rather than absolute suppression of dissociation [8,39]. Note that the dissociation rate of total TTR in plasma decreases as tafamidis concentration increases; however, it has not been specified quantitatively how various bound states of TTR contribute to this change [8].

The TTR concentration remains at a dynamic steady-state level governed by its synthesis and degradation processes. These processes are quantified by rates and can be described using a zero-order synthesis and first-order degradation turnover framework, as previously reported [9]. Under this formulation, TTR dynamics can be expressed as:

(1) $$\begin{array}{c} \frac{dTTR}{dt}\left[\mu M\right]=k_{synthesis}\left[\frac{\mu M}{\mathrm{day}}\right]-k_{degradation\;}\left[\frac{1}{\mathrm{day}}\right]\times TTR\;\left[\mu M\right], \end{array}$$

yielding a steady-state concentration of:

(2) $$\begin{array}{c} TTR_{baseline}\left[\mu M\right]=\frac{k_{synthesis}\left[\frac{\mu M}{\mathrm{day}}\right]}{k_{degradation}\left[\frac{1}{\mathrm{day}}\right]} \end{array}$$

Consequently, any change in the steady-state concentration of circulating TTR must arise from a change in the ratio between its synthesis and elimination rates. Let ${TTR}_{baseline}$ denote the baseline steady-state concentration prior to tafamidis administration, and ${TTR}_{ss,new}$ the steady-state concentration after drug saturation. An observed 33% increase corresponds to:

(3) $$\begin{array}{c} {TTR}_{ss,new}\left[\mu M\right]=1.33\times {TTR}_{baseline}\left[\mu M\right]. \end{array}$$

When monomer elimination is negligible, the steady-state tetramer concentration is governed by the balance between synthesis and degradation. In this framework, proportional scaling of either the synthesis rate or the degradation rate leads to mathematically identical steady-state solutions. Consequently, steady-state data alone cannot distinguish whether the observed increase in circulating TTR arises from enhanced synthesis or reduced elimination; these mechanisms bring structural non-identifiability to the model. This structural ambiguity is not specific to the present PBPK–QSP framework but reflects a general limitation of turnover models constrained solely by steady-state observations, as discussed previously in a phenomenological context [10].

To reproduce the observed population-average increase in steady-state TTR, we introduced a turnover-scaling factor applied to the effective degradation rate such that:

(4) $$\begin{array}{c} \frac{k_{synthesis}\;\left[\frac{\mu M}{\mathrm{day}}\right]}{s\times \;k_{degradation}\;\left[\frac{1}{\mathrm{day}}\right]}=1.33\times \frac{k_{synthesis}\;\left[\frac{\mu M}{\mathrm{day}}\right]}{k_{degradation}\;\left[\frac{1}{\mathrm{day}}\right]}, \end{array}$$

yielding the solution where s=1/1.33≈0.75.

Importantly, the scaling factor *s* is mathematically symmetric with respect to synthesis and degradation: it could equivalently be applied to the numerator or denominator of Equation (4) without altering model predictions. Consequently, steady-state TTR data constrain only the ratio of synthesis to degradation rates, and the parameter *s* should be interpreted as a turnover-scaling factor agnostic to whether the underlying physiological effect acts on synthesis or elimination. The present implementation on degradation therefore represents one hypothesis-consistent parametrization rather than a uniquely identifiable mechanistic attribution.

Tafamidis binds directly to circulating TTR tetramers and induces stabilizing conformational changes, which in the model are assumed to alter internalization or clearance pathways of the protein; this assumption is supported by the high fraction of circulating TTR present in the drug-bound state. In contrast, increased hepatic TTR synthesis is unlikely to underlie the observed rise in circulating TTR, as in vitro evidence supports post-translational modulation of secretion at the site of synthesis, requiring intracellular (endoplasmic reticulum) access; however, these findings do not necessarily translate quantitatively to the in vivo setting [40]. Accordingly, the QSP model is calibrated to a known population-level steady-state effect and is not intended to provide predictive discrimination between synthesis- and clearance-based mechanisms.

**Figure 1:**
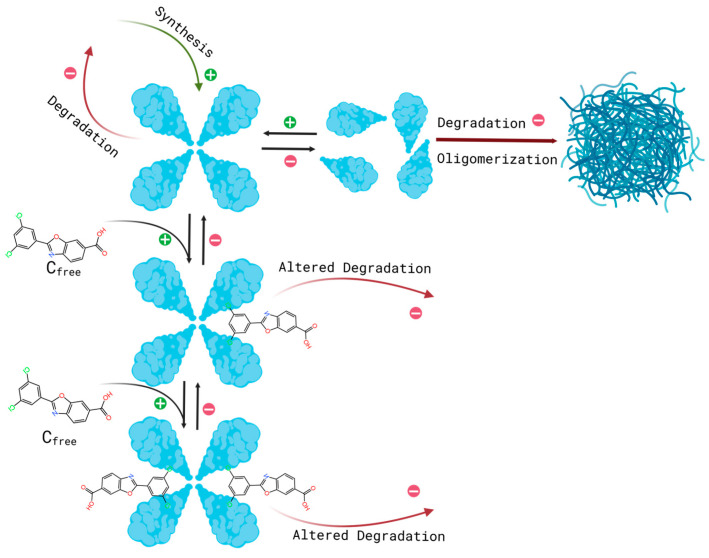
Schematic diagram of the PBPK–QSP linkage and QSP structure. Tafamidis plasma concentrations simulated by the PBPK model enter the QSP module as the time-varying total drug concentration, from which free tafamidis is computed based on competitive binding to transthyretin and albumin.

The main differences between the current and previously presented minimalistic QSP model include the application of two stable bound states of transthyretin and the introduction of a drug concentration variable as an input from the above-described PBPK model [9]. The previously presented minimalistic model was developed to provide a conceptual and mathematical framework for understanding TTR dynamics in vivo, with particular emphasis on disentangling the roles of tetramer stabilization, synthesis, and elimination. The model describes TTR as an open system with production, reversible tetramer–monomer interconversion, and irreversible elimination from the circulation. Under its core assumptions—rapid monomer–tetramer reassociation relative to monomer degradation—the model demonstrates analytically that changes in tetramer dissociation rate alone do not alter steady-state TTR concentration. Instead, clinically observed increases in circulating TTR can only be reproduced by concurrent changes in TTR production and/or elimination rates. Thus, the purpose of the minimal model development was mechanistic understanding to rationalize clinical observations and identify which physiological processes must be affected by tetramer stabilizers to explain increased serum TTR levels. The current work builds upon this framework by extending it to a mechanistic, state-resolved QSP model that explicitly represents multiple drug-bound TTR states, albumin binding, and dynamic tafamidis exposure provided by a PBPK model, thereby enabling quantitative simulation and prediction of clinically observed TTR responses. The general structure of the proposed model can be seen in Figure 1.

The analysis of drug effect relies on precise description of total TTR concentration (${TTR}_{total}$) in plasma, as it is the only surrogate of drug action that can be quantitatively tracked. To characterize the sensitivity of model-predicted TTR responses to tafamidis dose, a dose-sensitivity analysis was performed under steady-state conditions by systematically varying the administered tafamidis meglumine dose around the recommended clinical regimen (4 × 20 mg, corresponding to 48.8 mg tafamidis). Simulations were conducted for fractional and multiple doses equal to twofold, three-quarters, one-half, and one-quarter of the standard dose, selected to span a clinically relevant and practically divisible range. For each dose level, steady-state values of total, bound, free, and monomeric TTR species were extracted, with particular emphasis on the unbound-to-total-TTR ratio as an integrative, model-derived quantity summarizing dose-dependent changes in TTR binding at the population level.

We suggested a mechanism combining TTR degradation rate modification with molecular-level TTR binding. However, it should be noted that in some cases, steady-state TTR levels may increase through stabilization alone, without affecting degradation, although such increases are typically modest, not exceeding, on average, 15% [10].

The full model code, in the form of a notebook, together with parameter descriptions, can be found in the Supplementary Materials. We provide results for models with two different association and dissociation constants, coming from separate experiments, described in two articles; the code can be easily adjusted to switch from one set of parameters to another [37,39]. Results of the simulations are available as Excel files attached with the Supplementary Materials.

#### 2.3.1. QSP Model Parametrization

While a static model would be sufficient for monitoring steady-state concentrations, incorporation of dynamics enhances the model. It allows for tracking delays in effect and accounting for time-dependent variations in individual drug concentrations. A total of six ordinary differential equations (ODEs) were used in the model (see equations below). The QSP model parameters are categorized into two groups: drug-specific parameters and system (patient)-specific parameters. The latter, namely system parameters, were provided as individual values for virtual individuals as detailed in a subsequent section. After analysis of the binding affinities, two sets of association and dissociation rate constants were applied: one derived from the equilibrium binding constant reported in Nelson et al. and the other one directly adopted from Corazza et al. [37,39]. To account for the observed TTR dynamics, a fixed scaling factor was introduced to describe changes in the clearance of the drug–protein complex. Specifically, the model was calibrated using scaling factors of 0.75 for TTR1 and TTR2 clearances, which allows the model to predict an average increase of 33% in TTR plasma concentration in healthy male volunteers, as previously reported in the literature [6].

Identifiability of the synthesis and clearance scaling factors was evaluated using a local Fisher Information Matrix (FIM)-based analysis with a steady-state pseudo-observation reflecting the reported 33% increase in total TTR concentration. Results of the analysis demonstrated that synthesis and clearance scaling factors cannot be estimated independently using available data, exhibiting strong correlation and a rank-deficient FIM. Consequently, the attribution of the observed steady-state increase to reduced effective clearance represents a mechanistically motivated modeling assumption rather than a statistically identifiable fact. Full details of the identifiability analysis are provided in the Appendix A (R scripts).

Generative AI support (GPT-5.2 large language model, OpenAI; accessed January 2026) was used in a limited technical-assistance role during development of the Fisher Information Matrix identifiability workflow. Specifically, the model was used to help formalize the computational procedure for local FIM construction, including symbolic-to- numeric mapping of model parameters, structuring of finite-difference sensitivity calculations, and implementation logic for the steady-state pseudo-observation approach in R. The AI tool did not generate primary scientific results, select model assumptions, or interpret identifiability outcomes; all equations, parameterizations, scripts, and conclusions were defined, verified, and validated by the authors.

All parameters used for building the QSP model are presented in the Appendix A (see Table A2).

#### 2.3.2. Equations Describing Tafamidis–TTR Kinetics


(5) $$\begin{array}{c} \frac{dC_{TTR1}}{dt}=-T_{bound,deg}\times C_{TTR1}+k_{on1}\times C_{Taf}\times C_{TTRfree}-k_{off1}\times C_{TTR1}+k_{off2}\times C_{TTR2\;}-k_{on2}\times C_{Taf}\times C_{TTR1}\; \end{array}$$

The concentration of the single tafamidis–TTR complex increases when free transthyretin (${TTR}_{free}$) binds to a tafamidis molecule, initiating stabilization of TTR. The single tafamidis–TTR complex also rises when the double tafamidis–TTR complex dissociates, releasing one tafamidis molecule and reverting to the single-bound form. Conversely, the single tafamidis–TTR complex concentration decreases through three mechanisms: irreversible degradation of the complex, dissociation of tafamidis from TTR into their free forms, and the binding of an additional tafamidis molecule to the single complex, forming the double tafamidis–TTR complex.

In Equation (5), $k_{on1}$ represents the primary association rate of tafamidis with TTR ($\mu M^{-1}h^{-1}$), while $k_{off1}$ denotes the primary dissociation rate of tafamidis from TTR (1/h). The formation of the double complex is governed by $k_{on2}$, the secondary association rate of tafamidis with the single-bound complex ($\mu M^{-1}h^{-1}$), and its dissociation is described by $k_{off2}$ (1/h). The irreversible elimination is the same for single- and double-bound complexes, assigned as $T_{bound,deg}$ (1/h), which is scalar adjusted via $T_{deg}$.

(6) $$\begin{array}{c} \frac{dC_{TTR2}}{dt}=-T_{bound,deg}\times C_{TTR2}+k_{on2}\times C_{Taf}\times C_{TTR1}-k_{off2}\times C_{TTR2} \end{array}$$

The concentration of the double tafamidis–TTR complex increases when a second tafamidis molecule binds to the single tafamidis–TTR complex, but without accounting for enhanced resistance to dissociation. Conversely, this concentration decreases through two primary mechanisms: irreversible degradation of the double complex and dissociation of one tafamidis molecule, which converts the double complex back into the single-bound form.

In Equation (6), $k_{on2}$ represents the secondary association rate of tafamidis with the single-bound TTR complex ($\mu M^{-1}h^{-1}$), while $k_{off2}$ denotes the secondary dissociation rate of tafamidis from the double complex (1/h).

(7) $$\begin{array}{c} \frac{dC_{TTRfree}}{dt}=r+k_{a}\times {C_{Monomer}}^{4}-\left(k_{diss}+T_{deg}\right)\times C_{TTRfree}-k_{on1}\times C_{Taf}\times C_{TTRfree}+k_{off1}\times C_{TTR1}\; \end{array}$$

The concentration of free TTR increases through its natural synthesis in the body and the dissociation of tafamidis from the single tafamidis–TTR complex, releasing TTR back into its free form, as well as due to reassociation of monomers back to tetramers with rate $k_{a}$ ($\mu M^{-3}h^{-1}$). Conversely, free TTR is reduced by three key processes: natural degradation of TTR; dissociation into monomers, which reflects destabilization of its native tetrameric structure and increases the risk of aggregation; and binding with tafamidis to form the single tafamidis–TTR complex.

In Equation (7), r represents the rate of TTR synthesis (μM/h), while $T_{deg}$ denotes the natural degradation rate of TTR (1/h). The dissociation of TTR into monomers is described by $k_{diss}$ (1/h), and the binding of free TTR with tafamidis to form the single complex is governed by $k_{on1}$ ($\mu M^{-1}h^{-1}$). The reverse process, where tafamidis dissociates from the single complex, is described by $k_{off1}$ (1/h).

(8) $$\begin{array}{c} \frac{dC_{Monomer}}{dt}=4k_{diss}\times C_{TTR}-4\times k_{a}\times {C_{Monomer}}^{4}-M_{deg}\times C_{Monomer}\; \end{array}$$

The concentration of monomers increases when free TTR dissociates from its tetrameric form, reflecting destabilization of the native structure. This dissociation is a critical step in the pathogenesis of amyloid diseases, as monomers are prone to misfolding and aggregation into amyloid fibrils. Conversely, the monomer concentration decreases when monomers re-associate to form stable tetramers, restoring TTR’s functional structure; undergo irreversible degradation; or clump to aggregates. For the proposed model the last two are indistinguishable; thus, they are merged within a single parameter.

In Equation (8), $k_{diss}$ represents the dissociation rate of tetrameric TTR into monomers (1/h), while $k_{a}$ denotes the association rate of unbound TTR monomers to reform tetramers ($\mu M^{-3}h^{-1}$). The irreversible degradation of monomeric TTR is defined by $M_{deg}$ (1/h). Direct quantitative measurements of dissociated TTR monomers in plasma are limited; however, subunit-exchange analyses in human plasma indicate that monomeric species constitute well below 1% of total circulating TTR, providing an empirical order-of-magnitude constraint [41]. Accordingly, in the model the monomer concentration is derived from the steady-state balance between tetramer dissociation and reassociation kinetics (Appendix A, Table A2, Equation (A1)), yielding values consistent with these experimental plasma estimates.

(9) $$\begin{array}{c} \frac{dC_{Alb1}}{dt}=k_{on,albumin}\times C_{Taf}\times C_{Alb,free}-k_{off,albumin}\times C_{Alb1}\; \end{array}$$

Tafamidis can bind to albumin in the plasma, forming a tafamidis–albumin complex, thus reducing the availability of free tafamidis for transthyretin stabilization. This interaction serves as a reservoir, modulating the pharmacokinetics of tafamidis, mainly by keeping the fraction unbound at a stable level. Conversely, the number of tafamidis–albumin complexes decreases when tafamidis dissociates from albumin, releasing free tafamidis back into the blood.

In Equation (9), $k_{on,albumin}$ represents the association rate of tafamidis with albumin ($\mu M^{-1}h^{-1}$), while $k_{off,albumin}$ denotes the dissociation rate of tafamidis from the tafamidis–albumin complex (1/h).

(10) $$\begin{array}{c} \frac{dC_{Alb,free}}{dt}=-k_{on,albumin}\times C_{Taf}\times C_{Albumin}+k_{off,albumin}\times C_{Alb1}\; \end{array}$$

Albumin is present in abundance; however, it is necessary to track its concentration to capture all drug interactions. We assume that there is only one binding site of tafamidis to albumin and that only unbound albumin, described with Equation (10), is available for binding [11].

(11) $$\begin{array}{c} C_{Taf}=C_{total}-\left(C_{TTR1}+2\times C_{TTR2}+C_{Alb1}\right)\; \end{array}$$

The concentration of free tafamidis in plasma is determined by the total administered dose, excluding the amount bound to protein complexes. We assume that only the unbound drug is available to interact with TTR. Total concentration ($C_{total}$), mentioned in Equation (11), represents the total tafamidis concentration and is treated as the merging variable for the PBPK and QSP model. All concentrations are expressed in μM.

### 2.4. Population-Level Sampling of TTR Concentrations

To conduct simulations in a virtual population, baseline TTR concentrations were generated for thirty virtual healthy male subjects aged 30–40 years, using summary statistics reported by Ingenbleek et al. for age- and sex-stratified groups [42]. This age range was selected deliberately for methodological consistency. First, the only publicly available multiple-dose tafamidis pharmacokinetic data approaching steady state were generated in healthy male volunteers within this age range and were used to evaluate the PBPK component of the model [19]. Second, the most frequently cited human estimates of transthyretin elimination kinetics were derived from in vivo radiolabeled protein kinetic studies conducted in healthy males aged 30–40 years, as reported by Oppenheimer et al. and Socolow et al. [14,15]. These studies reported first-order degradation rate constants of approximately 0.363 day^−1^ (Oppenheimer et al.) and 0.268 day^−1^ (Socolow et al.). Ingenbleek et al. reported that baseline TTR concentrations within each age–sex stratum are approximately normally distributed [42]. Individual baseline TTR values were sampled randomly from a normal (μ,σ) distribution parameterized using the reported mean and standard deviation for this group. The number of virtual subjects (*n* = 30) was selected to match the size of the multiple-dose clinical pharmacokinetic study used to evaluate the PBPK component, thereby aligning simulated between-subject variability with the available observed study cohort [19]. One baseline value was assigned per virtual subject and held constant across simulations, thereby preserving the reported between-subject variability in baseline TTR concentrations.

Female subjects were not simulated in the present work. Although baseline TTR distributions by sex are available, the data required to parameterize and validate sex-specific tafamidis exposure under a multiple dosing regime and to quantitatively support transferability of the observed TTR increase across sexes are limited [42]. To avoid introducing additional unverifiable assumptions, the present analysis was restricted to males, and generalization of the results to female populations is acknowledged as a limitation.

Initial values for optimization were derived from Oppenheimer et al., as this study reports turnover estimates in a demographic group matching the selected virtual population [14]. Parameter fitting and population sampling were implemented using the R programming language. Full optimization results, sampling scripts, and associated code are provided in the Supplementary Material.

## 3. Results

The developed PBPK–QSP model effectively describes the overall drug effect on TTR and successfully replicates tafamidis pharmacokinetic outcomes observed in multiple-dose studies conducted in a healthy population. The comparison of observed and predicted PK parameters (AUC, $C_{max}$, $T_{max}$) is shown in Table 2.

**Figure 2:**
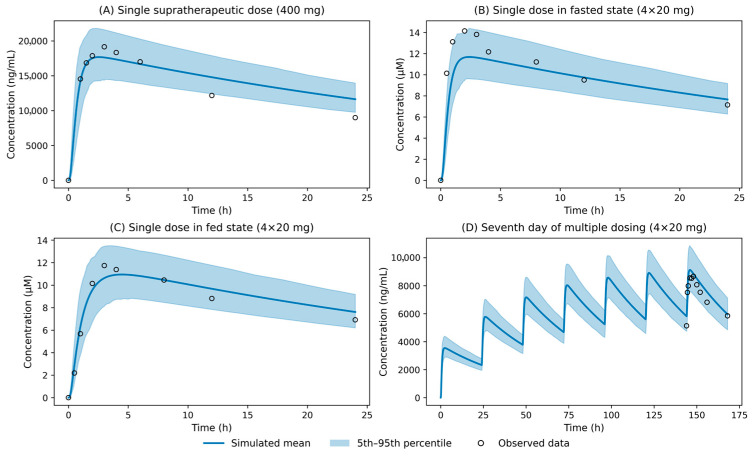
Observed clinical data (open circles) versus simulated mean concentration across 10 virtual trials (solid line), with the 5th–95th percentile range across virtual trials shown as a shaded area, following (**A**) a single supratherapeutic dose of tafamidis meglumine (400 mg), (**B**) a single dose administered in the fasted state (4 × 20 mg), (**C**) a single dose administered in the fed state (4 × 20 mg), and (**D**) the seventh day of multiple dosing (4 × 20 mg).

The pharmacokinetic model’s performance in predicting tafamidis exposures after multiple-dose therapy in a 30–40-year-old male population is presented in Figure 2D. Moreover, simulated tafamidis plasma concentration–time profiles following a single supratherapeutic 400 mg dose were consistent with observed clinical data in a cohort of 42 healthy individuals (Figure 2A). The model also successfully captured the pharmacokinetics of single-dose administration in 16 healthy volunteers enrolled in trial B3461054, demonstrating robust predictive performance under both fasted and fed conditions (Figure 2B,C) [17]. Across all evaluated dosing regimens and nutritional conditions, predicted exposure metrics (AUC and $C_{max}$) showed good agreement with observations, with individual fold errors ranging from 0.74 to 1.12. Based on geometric mean AUC and $C_{max}$ values, the overall AFE was 0.93, indicating a slight underprediction, while the AAFE was 1.13, corresponding to a typical prediction error of approximately 13%.

**Figure 3:**
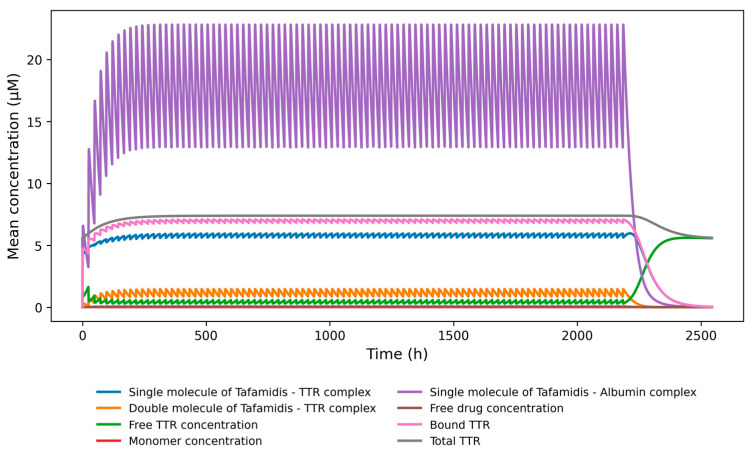
Results of simulation of tafamidis binding, accounting for dissociation rates of drug from Nelson et al. 2021 [39].

**Figure A2:**
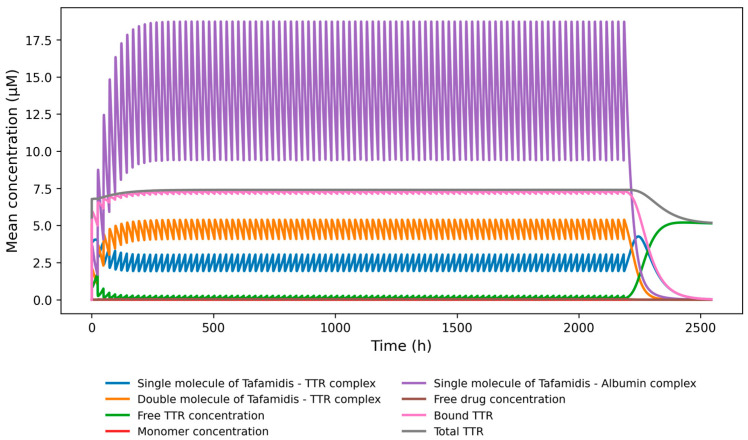
Tafamidis binding when dissociation constants from Corrazza et al. 2019 [37] are applied. In this scenario, we observe much higher double-bound tafamidis–TTR complex concentration when compared to simulation with the applied Nelson et.al. 2021 constants [39].

Figure 3 illustrates the distribution of bound tafamidis across the single- and double-bound states of TTR, as well as its interaction with albumin, which acts as a buffering pool for circulating tafamidis and modulates its availability for TTR binding. Comparison of results obtained using the Nelson-based and Corazza-based binding parameter sets shows that the former yields a higher proportion of single-bound TTR–tafamidis complexes, whereas the latter shifts the distribution toward a higher abundance of double-bound complexes (Figure 3 and Figure A2).

**Figure 4:**
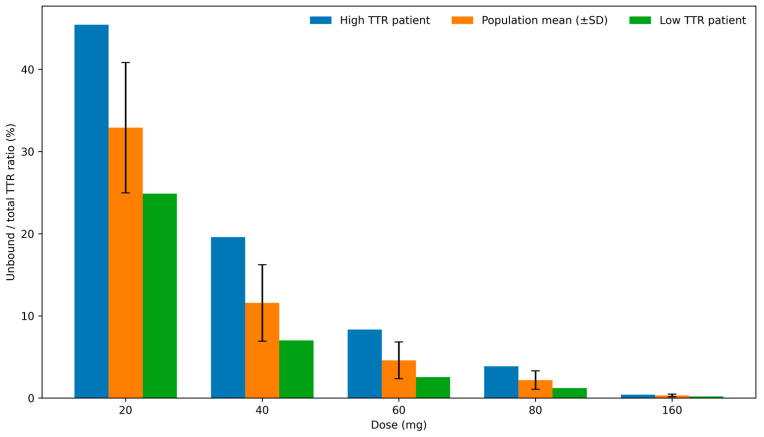
Unbound-to-total-TTR dose-sensitivity analysis. Whiskers represent standard deviation.

**Figure A3:**
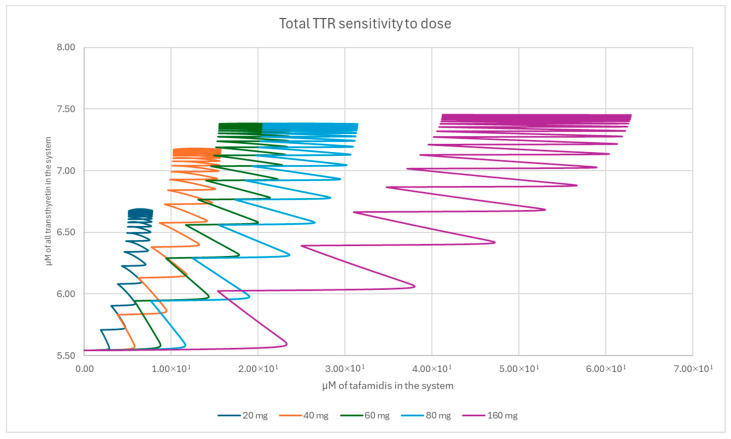
Total TTR sensitivity to dose.

**Figure A4:**
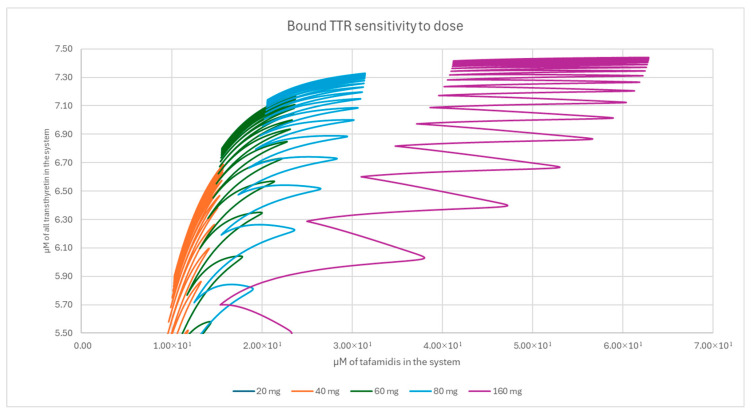
Bound TTR sensitivity to dose.

**Figure A5:**
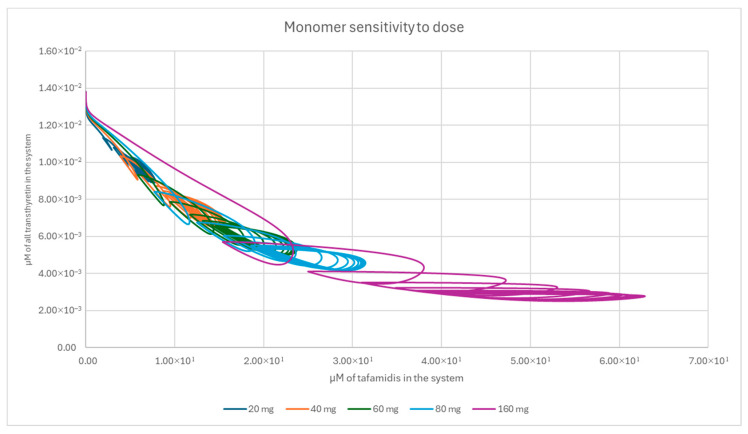
Monomer sensitivity to dose.

**Figure A6:**
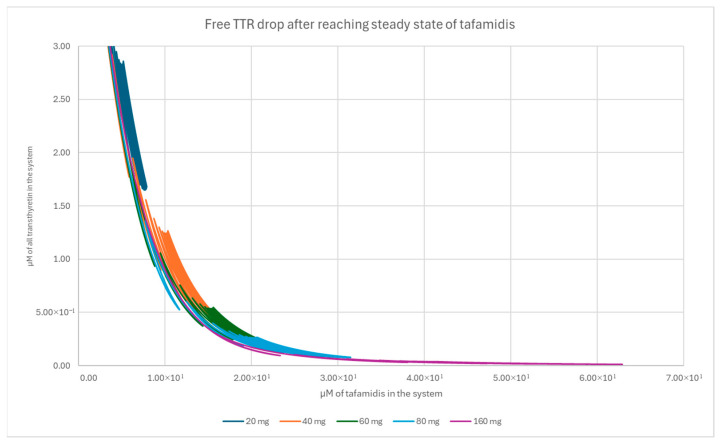
Free TTR sensitivity to dose.

The steady-state unbound-to-total-TTR ratio exhibited a clear dependence on tafamidis dose across the evaluated range. Relative to the recommended tafamidis meglumine regimen (4 × 20 mg; 48.8 mg tafamidis), stepwise dose reductions to three-quarters, one-half, and one-quarter of the standard dose were associated with progressively higher unbound-to-total-TTR ratios, whereas a twofold dose increase resulted in a further reduction in the unbound fraction. The predicted dose–response relationship for the unbound-to-total-TTR ratio is shown in Figure 4. Dose-dependent changes in total, bound, free, and monomeric TTR species are presented in the Appendix A (Figure A3, Figure A4, Figure A5 and Figure A6).

## 4. Discussion

Tafamidis has been approved for the treatment of transthyretin amyloid cardiomyopathy based on demonstrated clinical benefit, including improved survival and reduced cardiovascular-related hospitalizations, together with strong experimental evidence showing its ability to kinetically stabilize transthyretin tetramers in vitro and ex vivo. The present modeling work does not aim to reproduce or predict clinical outcomes. Instead, it builds on the established mechanism of action of tafamidis—namely, stabilization of the transthyretin structure.

The presented PBPK–QSP model successfully replicates tafamidis pharmacokinetics in a healthy population, supporting its applicability for dose selection and clinical trial optimization. Tafamidis is a long-term therapy, and accordingly, the integrated PBPK–QSP model simulation was run over a sufficiently long-term horizon to reach pharmacodynamic steady state. It should be noted that pivotal clinical evidence for tafamidis efficacy derives predominantly from elderly ATTR patients, often with multiple comorbidities. Age- and disease-related physiological changes may affect both tafamidis pharmacokinetics and TTR turnover; therefore, extrapolation of simulation results from young healthy males to the clinical ATTR population should be done cautiously. In the absence of age-matched longitudinal data suitable for parameterization and validation within this framework, the reported average ~33% increase in total TTR concentration was used as a calibration target, and this assumption is explicitly treated as a limitation of the model. Moreover, the model accurately predicts tafamidis plasma concentrations following a single supratherapeutic 400 mg dose (Figure 2A), supporting its applicability for dose selection.

On the PD side, the model captures trends in average TTR concentration changes; however, its predictive performance cannot currently be evaluated at the individual level. This is because the model was developed based on the data derived from population-level analysis. If the individual TTR trajectories were available, the model’s predictive accuracy for patient-specific responses could be assessed and further refined. The variability in TTR concentration appears to depend on the baseline protein levels and overall patient health, but the precise contributing factors remain unidentified. Addressing this limitation could potentially enhance the model’s predictive accuracy and increase its usefulness towards personalized medicine. Our analysis indicates that patients with low baseline TTR levels may achieve adequate stabilization at lower doses. For instance, a low-TTR patient (3.85 μM) dosed with 60 mg tafamidis meglumine reached steady-state fractional stabilization of 2.55%, comparable to the population average of 2.19%. In contrast, a high-TTR patient (7.51 μM) required 80 mg daily to reach 3.86% stabilization, a value close to that achieved by the average patient on 60 mg (4.59%). The dose levels in this analysis were chosen deliberately: while the standard regimen involves four 20 mg tablets, reducing the dose to three tablets in patients with low TTR could lower treatment costs without compromising therapeutic effect. Conversely, patients with high baseline TTR may require higher doses to achieve optimal stabilization. These simulations suggest that baseline TTR concentration may influence the exposure–stabilization relationship; however, any dose individualization based on baseline TTR would require prospective clinical validation and should be regarded as hypothesis-generating.

Dose-sensitivity analysis has shown that there is no clear difference in the binding effect across the tested dose range (Figure A3, Figure A4, Figure A5 and Figure A6). However, the simulations suggest that baseline TTR may influence the exposure–stabilization relationship in extreme cases; this observation is hypothesis-generating and would require prospective clinical validation before informing dosing decisions. Patients with lower TTR levels could potentially decrease the dose or extend dosing gaps. A critical aspect of the model’s implementation is the necessity of applying a fixed scaling factor to describe changes in drug–protein complex clearance. Due to the lack of sufficient experimental data, this heuristic adjustment was required to align predictions with observed clinical data. Similarly, the rate of monomer degradation was set to a constant value in the absence of experimental data on its actual magnitude. This choice is justified by the fact that, under physiological conditions, the product of k_association × M³ is expected to be substantially lower than k_deg,monomer × M, given that monomer concentrations are below 1% of tetramer concentrations (as reported by Sekijima et al. 2001) [41]. Consequently, variations in this parameter are unlikely to dominate system dynamics; however, this assumption should be revisited if future data indicate otherwise.

A key methodological point is that, in an open turnover system such as circulating transthyretin, a clinically observed steady-state increase in total TTR can generally be reproduced by multiple mechanistic hypotheses that act on different parts of the turnover balance. In particular, reduced tetramer dissociation, altered effective clearance/internalization of stabilized species, and modulation of net production can lead to similar steady-state outcomes, rendering the underlying mechanism structurally ambiguous when only steady-state endpoints are available [10]. In this light, the present PBPK–QSP framework is intended not as definitive mechanistic proof, but as a quantitative scaffold that preserves mechanistic structure where parameters are experimentally constrained (tafamidis exposure, binding stoichiometry and kinetics), while representing unresolved biology through a minimal number of calibrated terms. This modeling stance allows for the integration of pharmacokinetic information with TTR turnover physiology and provides a principled way to constrain the space of plausible mechanisms consistent with observed PK and population-average TTR responses, thereby guiding which future measurements would be most informative for discriminating between competing hypotheses.

The proposed model does not uniquely identify the biological mechanism underlying the observed increase in TTR concentrations but constrains which turnover and clearance hypotheses remain consistent with the available steady-state and pharmacokinetic data. Future research should therefore focus on elucidating whether this phenomenon is driven by modified clearance, ligand-dependent internalization of bound complexes, or potential effects of tafamidis on TTR synthesis rates. Quantitative information is currently lacking on how ligand binding alters endothelial transport, tissue uptake, and intracellular internalization or degradation of TTR–ligand complexes, limiting mechanistic attribution within the present framework. Additional studies are also needed to clarify albumin’s competitive role in tafamidis binding and its impact on pharmacokinetics.

More broadly, the PBPK–QSP model illustrates how mechanistic pharmacology models can identify which physiological processes must be quantified to resolve competing hypotheses, while indicating that other system components are already sufficiently constrained by existing data. While the model demonstrates strong predictive capability at the pharmacokinetic and steady-state TTR level, further refinements and validation studies are required to fully capture the biological determinants of tafamidis–TTR interplay.

## Figures and Tables

**Table 1:** Tafamidis clinical data and study characterization.

Study/Trial Identifier	Study Purpose and Design	Dose Regimen and PK Data Used in This Work
Thorough QT study (NCT01775761) [16]	Phase I; randomized crossover; healthy volunteers (*n* = 42); fasted; QTc evaluation with PK as secondary endpoint.	Single oral supratherapeutic dose of tafamidis meglumine (400 mg); observed plasma PK (0–24 h); mean concentration–time data used to evaluate high-exposure behavior.
Single-dose relative bioavailability and food-effect study (B3461054) [17,18]	Phase I; open-label; randomized 4-period, 4-sequence crossover; healthy volunteers (*n* = 16); comparison of free acid and meglumine formulation; comparison of fasted and fed conditions.	Single oral dose of tafamidis meglumine 80 mg (4 × 20 mg); observed plasma PK under fasted and fed conditions; mean concentration–time profiles used to evaluate food effect.
Phase Ib bioequivalence study (B3461056-Lockwood et al., 2020) [19]	Phase I; open-label; randomized crossover; healthy male volunteers (*n* = 30); fasted; bioequivalence study.	Tafamidis meglumine 80 mg (4 × 20 mg) administered once daily for 7 days; observed steady-state plasma PK; mean concentration–time profiles on day 7 used to evaluate multiple-dose behavior .

**Table 2:** Observed and PBPK-predicted pharmacokinetic parameters of tafamidis meglumine across multiple dosing regimens and nutritional conditions, with corresponding fold errors.

Study/Scenario	Dosing Regimen	State	PK Parameter	Statistic	Observed	Predicted	Fold Error (Pred/Obs)
**B3461056**	Multiple dose, 80 mg (4 × 20 mg), day 7	Fasted	AUC144–168 h (ng × h/mL)	Mean (SD)	169,600 (35,637)	180,557 (20,998)	1.06
			AUC144–168 h (ng × h/mL)	Median (range)	171,000 (125,000–258,000)	179,721 (127,744–254,519)	1.05
			AUC144–168 h (ng × h/mL)	Geometric mean (%CV)	166,200 (20)	179,362 (12)	1.08
			$C_{max}$ (ng/mL)	Mean (SD)	9241 (1796)	9160 (1039.26)	0.99
			$C_{max}$ (ng/mL)	Median (range)	8950 (6580–14,600)	9085 (6819.28–12,840.15)	1.02
			$C_{max}$ (ng/mL)	Geometric mean (%CV)	9087 (18)	9102 (11)	1.00
			$T_{max}$ (h)	Median (range)	2.0 (0.5–6.0)	1.90 (1.18–3.84)	0.95
**B3461054**	Single dose, 80 mg (4 × 20 mg)	Fasted	AUC0–inf (ng × h/mL)	Geometric mean (%CV)	203,400 (18)	188,274 (12)	0.93
		Fasted	$C_{max}$ (ng/mL)	Geometric mean (%CV)	4835 (20)	3586 (12)	0.74
		Fasted	$T_{max}$ (h)	Median (range)	1.5 (0.5–4.05)	2.26 (1.33–4.53)	1.51
		Fed	AUC0–inf (ng × h/mL)	Geometric mean (%CV)	208,100 (23)	188,505 (12)	0.91
		Fed	$C_{max}$ (ng/mL)	Geometric mean (%CV)	4132 (15)	3446.93 (12)	0.83
**NCT01775761**	Single dose, 400 mg (supratherapeutic dose study)	Fasted	$C_{max}$ (ng/mL)	Geometric mean (%CV)	20,360 (18)	17,659 (13)	0.87
		Fasted	AUC0–24 h (ng × h/mL)	Geometric mean (%CV)	305,400 (15)	342,313 (13)	1.12
		Fasted	$T_{max}$ (h)	Median (range)	2.0 (1–6)	2.3 (1.37–5.07)	1.15

## Data Availability

The original contributions presented in this study are included in the article/supplementary material. Further inquiries can be directed to the corresponding author(s).

## Supplementary Materials

The following supporting information can be downloaded at: https://www.mdpi.com/article/10.3390/pharmaceutics18030367/s1, PBPK–QSP results, QSP Lua code, population sampling R script with necessary data.

## Abbreviations

The following abbreviations are used in this manuscript:

AAFE	Absolute Average Fold Error
ADAM	Advanced Dissolution, Absorption, and Metabolism
AFE	Average Fold Error
AUC	Area Under the Curve
ATTR	Transthyretin Amyloidosis
BCRP	Breast Cancer Resistance Protein
BCS	Biopharmaceutics Classification System
$C_{max}$	Maximum Plasma Concentration
FDA	Food and Drug Administration
GIT	Gastrointestinal Tract
$k_{a}$	Association Rate Constant
$k_{diss}$	Dissociation Rate Constant
$k_{on}$	Association Rate Constant
$k_{off}$	Dissociation Rate Constant
$M_{deg}$	Monomer Degradation Rate Constant
PBPK	Physiologically Based Pharmacokinetics
PD	Pharmacodynamics
$P_{eff}$	Effective Permeability
$P_{app}$	Apparent Permeability
PK	Pharmacokinetics
QSP	Quantitative Systems Pharmacology
SD	Standard Deviation
So	Intrinsic Solubility
$T_{deg}$	TTR Degradation Rate Constant
$T_{max}$	Time to Maximum Concentration
TTR	Transthyretin
UGT	Uridine 5′-Diphospho-Glucuronosyltransferase
Vss	Volume of Distribution at Steady State

## Appendix A

**Table A1:** Drug input parameters for the PBPK model of tafamidis meglumine.

Parameter	Value	Origin of Data	Reference
**Physicochemical properties and blood binding**			
**Type of compound**	Acid	Based on structure	-
**MW, free acid (g/mol)**	308.12	Based on structure	-
**LogP**	3.91	Predicted with ALOGPS	[43]
**pKa**	3.7	-	[21]
**PSA (Å^2^)**	63.3	Predicted	-
**Blood and plasma binding**			
**Blood-to-plasma ratio (B:P)**	0.55	Methodological details unknown. Derived from regulatory documents.	[18]
**Fraction unbound in plasma (fu_p_)**	0.008	Experimental	[18]
**Absorption (solubility and permeability)** **Advanced Dissolution, Absorption and Metabolism model (ADAM)**			
**fu_gut_**	0.67393	Predicted	[33]
**Caco-2 apparent permeability (P_app_) (10^−6^ cm/s)**	30.9	Experimental	[24]
**Metoprolol permeability as a reference for Caco-2 experiment (P_app_) (10^−6^ cm/s)**	15.5	Experimental	[24]
**Effective permeability in human intestine (10^−4^ cm/s)**	6.9706	Predicted	[25]
**Solubility at pH 6.8 (mg/mL)**	3.121	Experimental	[26]
**Intrinsic solubility S_0_ (mg/mL)**	0.0024771	Predicted with Henderson–Hasselbach equation using solubility at pH 6.8 as an input.	[44]
**Distribution**			
**Volume of distribution at steady state**	0.16909 L/kg	Predicted with Simcyp population data, B:P and tissue-to-plasma partition coefficients.	[31,45]
**Tissue to plasma partition coefficients (K_p_)**			
**Adipose**	0.027	Obtained from rats. Rats distribution of small molecules is expected to be similar to that of humans.	[24]
**Brain**	0.022		
**Gut**	0.254		
**Heart**	0.113		
**Kidney**	0.59		
**Liver**	4.01		
**Lung**	0.107		
**Spleen**	0.063		
**Bone**	0.10203	Predicted using the Rodgers and Rowland method.	[45]
**Pancreas**	0.06316		
**Muscle**	0.036922		
**Skin**	0.28047		
**Additional organ**	0.039244		
**Elimination**			
**Described as a total clearance = 0.262 L/h. Tafamidis is eliminated mainly through glucuronidation in the liver. Unfortunately, there are not enough experimental data shared for bottom-up clearance prediction.**			

**Table A2:** Parameters used for building the QSP model.

Parameter Name	Value	Unit	Source
**TTR synthesis rate**	Patient-specific	μM/h	Based on Ingenbleek et al. dataset, as explained in “Population-level sampling of TTR concentrations” section [9,14,42].
**TTR degradation rate**	Patient-specific	1/h	Based on Ingenbleek et al. dataset, as explained in “Population-level sampling of TTR concentrations” section [9,14,42].
**Dissociation rate of unbound TTR**	0.0024	1/h	[8]
**Association rate of unbound TTR**	360,000	$\frac{1}{{\mu M}^{3}\cdot h}$	[46]
**Monomer degradation rate**	0.016	1/h	There are no data on this parameter available in the literature. We assume that for healthy volunteers it is the same as the average degradation rate of TTR [14].
**Association rate constant for first tafamidis–TTR binding**	16,200	$\frac{1}{\mu M\cdot h}$	[37]
**Dissociation rate constant for first tafamidis–TTR binding**	50.2 OR 28.8	1/h	Two alternative values presented: one fitted using KD from Nelson et al. (2020) combined with kon from Corazza et al. (2019); the other directly using koff from Corazza et al. (2019) [37,39].
**Association rate constant for second tafamidis–TTR binding**	12,600	$\frac{1}{\mu M\cdot h}$	[37]
**Dissociation rate constant for second tafamidis–TTR binding**	2998.8 OR 216	1/h	Two alternative values presented: one fitted using KD from Nelson et al. (2020) combined with kon from Corazza et al. (2019); the other directly using koff from Corazza et al. (2019) [37,39].
**Association rate constant for tafamidis–albumin binding**	1000	$\frac{1}{\mu M\cdot h}$	Exact value unknown. Determined by simultaneous fitting with the albumin–tafamidis complex dissociation rate to achieve KD=kdiss/ka [39].
**Dissociation rate constant for tafamidis–albumin binding**	1800	1/h	Exact value unknown. Determined by simultaneous fitting with the albumin-tafamidis complex association rate to achieve KD = kdiss/ka [39].
**Individual albumin concentration**	Patient-specific	μM	Albumin concentration calculated using a baseline (50.34 g/L) adjusted for age (coefficient = −0.0575) and BMI (coefficient = −0.0738), with an inter-individual coefficient of variation (CV) of 10%.
**Irreversible elimination rate of bound TTR–tafamidis complexes**	$T_{\deg}\;\times$ 0.75	1/h	Analytically solved for ~33% increase in total TTR concentration.

(A1)
$$\begin{array}{c} M_{st}=\sqrt[{4}]{\left(\frac{k_{diss}}{k_{a}}\times \frac{r}{\gamma }\right)\;} \end{array}$$

Equation (A1) defines baseline steady state of monomer concentration, where $k_{diss}$ and $k_{a}$ are the dissociation and association rates constants of TTR, r is the TTR synthesis rate, and γ is the degradation rate.
